# Effect of *CTLA-4* gene polymorphisms on long-term kidney allograft function in Han Chinese recipients

**DOI:** 10.18632/oncotarget.8714

**Published:** 2016-04-12

**Authors:** Yifeng Guo, Junwei Gao, Shuai Gao, Minghua Shang, Fang Guo

**Affiliations:** ^1^ Department of Urology, Shanghai General Hospital, Shanghai Jiao Tong University School of Medicine, Shanghai, China; ^2^ Department of Clinical Pharmacy, Shanghai General Hospital, Shanghai Jiao Tong University School of Medicine, Shanghai, China; ^3^ Department of Nephrology, Shanghai General Hospital, Shanghai Jiao Tong University School of Medicine, Shanghai, China; ^4^ Key Laboratory of Systems Biomedicine (Ministry of Education) and Shanghai Center for Systems Biomedicine, Shanghai Jiao Tong University, Shanghai, China

**Keywords:** kidney transplant, long-term allograft function, estimated glomerular filtration rate, cytotoxic T lymphocyte associated antigen-4, gene polymorphism, Pathology Section

## Abstract

Single nucleotide polymorphisms (SNPs) of cytotoxic T lymphocyte associated antigen-4 gene (*CTLA-4*) have been associated with graft rejection and long-term clinical outcome after organ transplantation. Our aim was to examine the association between *CTLA-4* SNPs (rs733618, rs4553808, rs5742909, rs231775, rs3087243) and long-term allograft function in Chinese renal transplant recipients. Genotyping of *CTLA-4* SNPs was performed in 292 renal transplantation recipients. To assess long-term allograft function, the estimated glomerular filtration rate (eGFR) was determined 1, 3, 6, 12, 24, 36, 48 and 60 months after renal transplantation. *CTLA-4* rs733618 and rs3087243 alleles and genotypes as well as the rs5742909 and rs231775 genotypes were significantly associated with long-term allograft function after transplantation (*P*<0.05). Patients with favorable genotypes had higher allograft function during the 60 months after transplantation. The TACGG, CACAG and CGTAA haplotypes were also associated with long-term kidney function after renal transplantation (*P*<0.05 or *P*<0.01). In sum, the favorable *CTLA-4* rs5742909TT genotype, *CTLA-4* rs733618C and rs3087243A alleles, and CACAG and CGTAA haplotypes, as well as the unfavorable *rs*733618TT, rs3087243GG and rs231775GG genotypes and TACGG haplotype could potentially serve as effective indicators of long-term allograft function in Chinese renal transplantation recipients.

## INTRODUCTION

In addition to generating a large repertoire of T cells that recognize a wide array of unknown pathogens, the thymus also adds self-reactive T cells to the peripheral T cell pool. Cytotoxic T lymphocyte associated antigen-4 (CTLA-4) is a key player in the control of self-reactive T cells. Genetic deficiency in CTLA-4 is lethal in mice due to profound immune dysregulation and autoimmune disease [1]. This highlights the critical immunoregulatory function of CTLA-4.

*CTLA-4* gene is located on chromosome 2q33. Single nucleotide polymorphisms (SNPs) in *CTLA-4* are associated with autoimmune diseases such as autoimmune thyroid disease and multiple sclerosis, and play an influential role in graft rejection and the long-term clinical outcome of organ transplantation [2–12]. The +49A/G (rs231775) SNP in exon 1 of *CTLA-4* results in a thr17-to-ala (T17A) substitution [13]. The −1772T/C (rs733618), −318C/T (rs5742909) and −1661A/G (rs4553808) SNPs are all located in the *CTLA*-4 promoter region and affect the expression of CTLA-4 and cause abnormal alternative splicing [14, 15]. The CT60G/A (rs3087243) SNP is a 3’ prime UTR polymorphism and encodes either a protective A/A genotype or a G/G genotype that predisposes the carrier to autoimmune disease [16].

We recently published two papers on the association between *CTLA-4* SNPs and the rate of acute rejection (AR) after renal transplantation [9, 10]. In the present study, we report that by estimating the glomerular filtration rate (eGFR) at different time intervals after renal transplantation, we determined that association between *CTLA-4* SNPs (*CTLA-4* rs733618, rs4553808, rs5742909, rs231775, rs3087243) and long-term renal function in Han Chinese transplant recipients.

## RESULTS

### CTLA-4 genotype and eGFR

**Figure 1:**
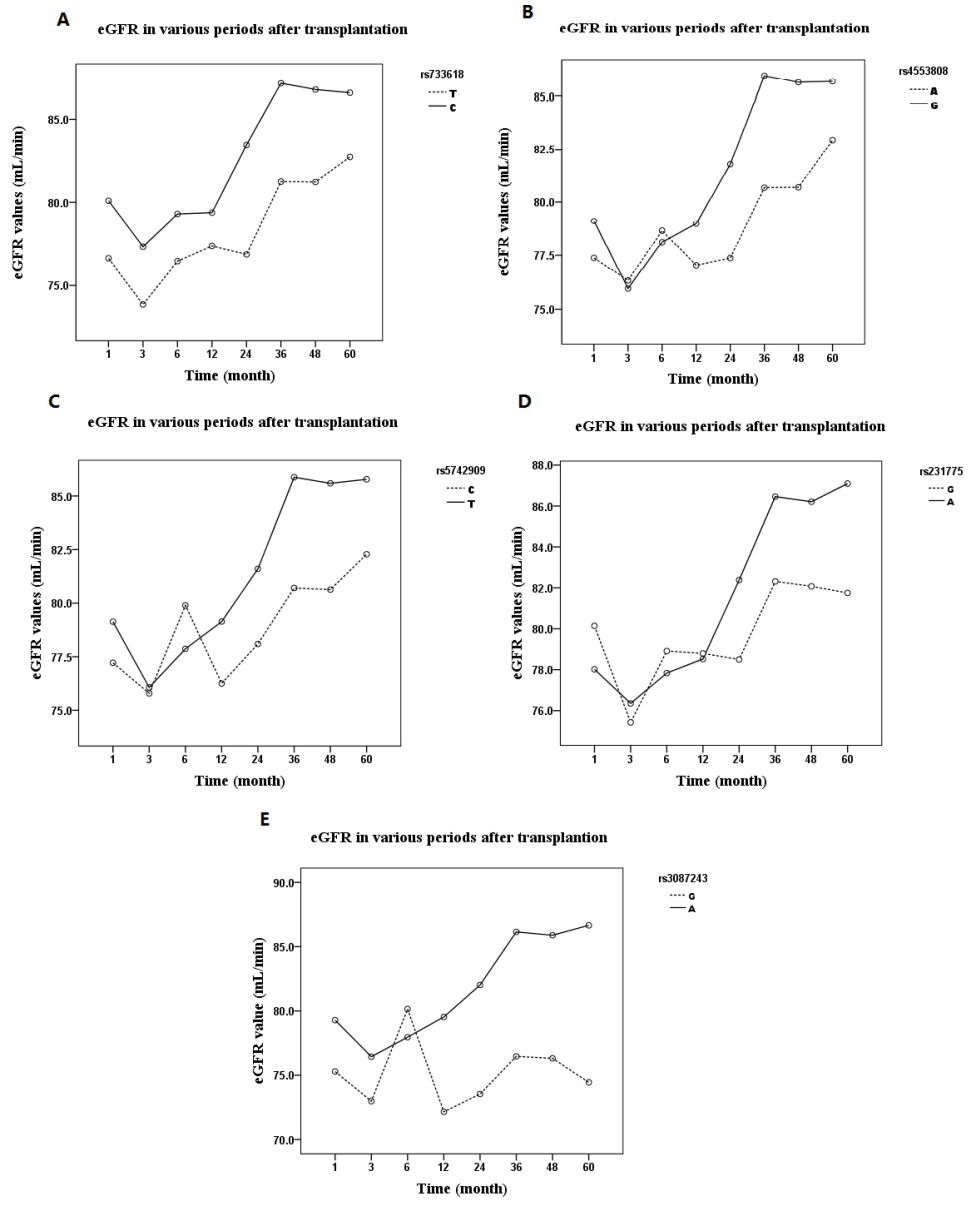
The influences of a11ele distribution of CTLA-4 SNPs on allograft function expressed as eGFR (estimated glomerular filtration rate) over 60 months: After renal transplantation, the eGFR trended upward in all allele groups (*P* < 0.05 in tests of within-subjects effects and multivariate analysis) **A.** rs733618, patients with C allele had better allograft function than those with T allele (*P* < 0.05 in test of between-subjects analysis); **B.** rs4553808; **C.** rs5742909; **D.** rs231775; **E.** rs3087243, patients with A allele had better allograft function than those with G allele (*P* < 0.05 in test of between-subjects analysis).

**Figure 2:**
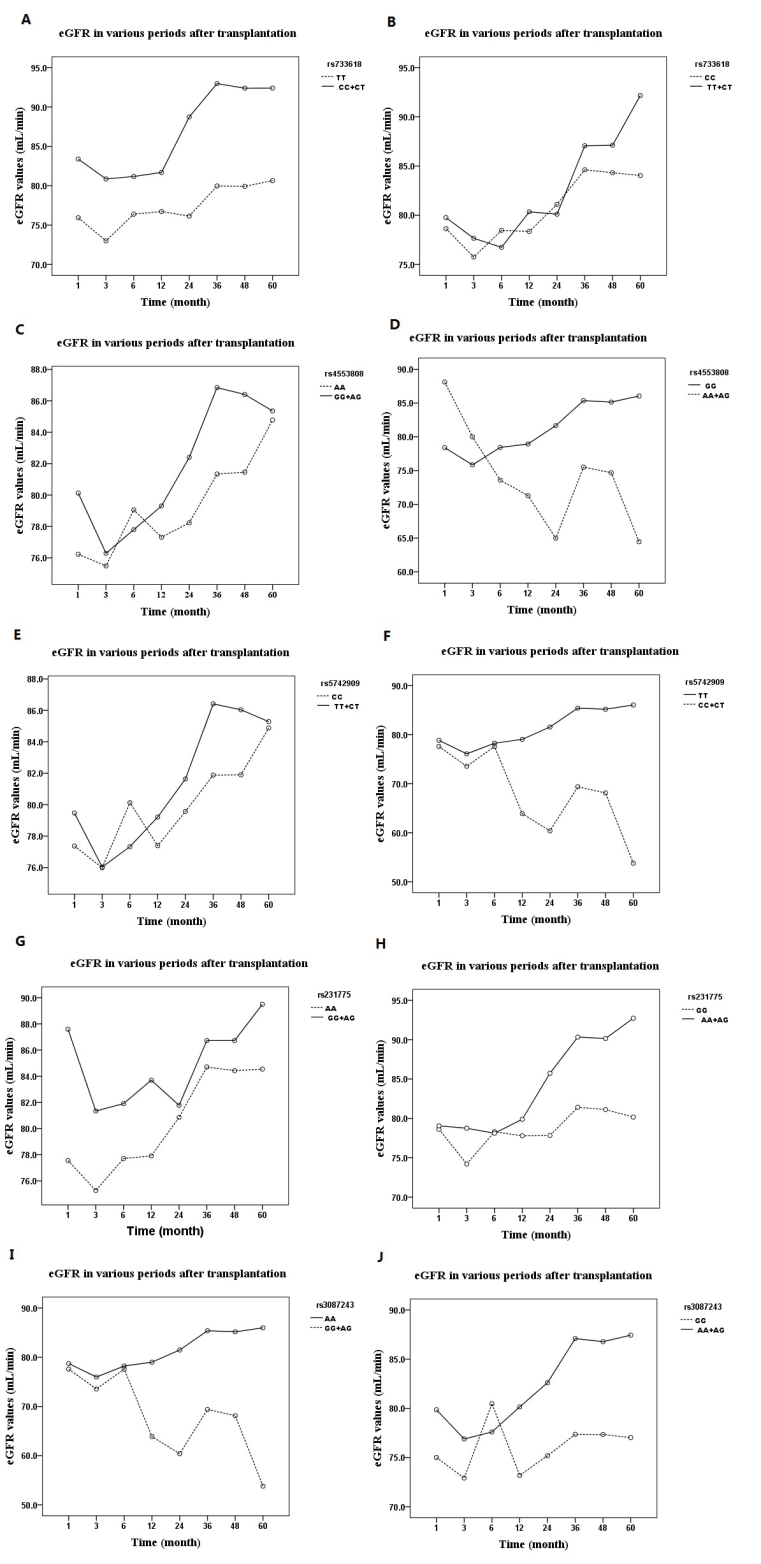
The influences of genotype distribution of CTLA-4 SNPs on allograft function expressed as eGFR over 60 months **A.** dominant effect of rs733618; **B.** Recessive effect of rs733618; **C.** dominant effect of rs4553808; **D.** Recessive effect of rs4553808; **E.** dominant effect of rs5742909; **F.** Recessive effect of rs5742909; **G.** dominant effect of rs231775; **H.** Recessive effect of rs231775; **I.** dominant effect of rs3087243; **J.** Recessive effect of rs3087243. eGFR: estimated glomerular filtration rate.

We examined the relations between *CTLA-4* SNPs (rs733618, rs4553808, rs5742909, rs231775 and rs3087243) and eGFR (eGFR < 90 mL/min/1.73 m^2^ was identified as renal failure and was staged according to the KDIGO guidelines) over a period of 60 months in recipients of kidney transplants. As shown in Figure 1 and Table 1, the C allele of rs733618 (220/584) and A allele of rs3087243 (72/584) were significantly associated with higher eGFR (*P <* 0.01). After renal transplantation, the eGFR trended upward in all allele groups (*P* < 0.05 in tests of within-subjects effects and multivariate analysis), but especially in the favorable allele groups (*P* < 0.05 in tests of between-subjects analysis). As shown in Figure 2 and Table 2, the dominant rs733618 genotype (TT/(CC+CT) (112/(40+140))) was significantly associated with the eGFR through the 60 months of follow-up (*P* < 0.05). At the same time, recessive analysis showed that rs5742909 (TT/(CC+CT) (8/(198+86))), rs3087243 (GG/(AA+AG) (228/(8+56))) and rs231775 (GG/(AA+AG) (116/(36+140))) were also related to eGFR (*P* < 0.05 in tests of between-subjects effects). Furthermore, for 60 months following the transplant operation, the eGFR showed opposite trends depending on whether the recipient had a protective genotype or one predisposing them to rejection (*P* < 0.05 in tests of within-subject effects and multivariate analysis).

**Table 1:** The influences of allele distribution of CTLA-4 SNPs on long-term allograft function over 60 months

Allele		Frequency	*P*-value		
			G	T	G*T
rs733618	T	364	0.008	0.000	0.019
	C	220			
rs4553808	A	475	0.209	0.000	0.050
	G	109			
rs5742909	C	472	0.215	0.000	0.031
	T	112			
rs231775	A	212	0.248	0.000	0.015
	G	372			
rs3087243	A	72	0.005	0.000	0.000
	G	512			

G = group; T = time; G*T = group*time interaction

**Table 2:** The influences of genotype distribution of CTLA-4 SNPs on long-term allograft function over 60 months

Genotype		Frequency	Model	*P*-value		
				G	T	G*T
rs733618	TT	112	Dominant	0.000	0.000	0.002
	CC	40	Recessive	0.543	0.000	0.082
	TC	140				
rs4553808	AA	192	Dominant	0.268	0.000	0.026
	GG	12	Recessive	0.198	0.073	0.004
	AG	88				
rs5742909	CC	198	Dominant	0.515	0.000	0.044
	TT	8	Recessive	0.049	0.000	0.049
	CT	86				
rs231775	AA	36	Dominant	0.176	0.001	0.299
	GG	116	Recessive	0.012	0.000	0.000
	AG	140				
rs3087243	AA	8	Dominant	0.050	0.021	0.011
	GG	228	Recessive	0.019	0.000	0.000
	AG	56				

G = group; T = time; G*T = group*time interaction

### CTLA-4 haplotype and eGFR (Table 3)

**Table 3:** Analysis of Haplotype Associations of CTLA-4 SNPs with long-term allograft function expressed as eGFR in different time intervals

Haplotype	Frequency (%)															
	1m		3m		6m		12m		24m		36m		48m		60m	
eGFR(ml/min/1.73 m^2^)	< 90	≥90	< 90	≥90	< 90	≥90	< 90	≥90	< 90	≥90	< 90	≥90	< 90	≥90	< 90	≥90
TACGG	59.0	57.9	49.8	59.6	58.5	58.1	60.5	57.7	63	56.4	67.5	52.7^**^	67.3	52.5^**^	66.9	53.7^**^
CACAG	13.3	16.6	11.9	16.3	17.3	15.4	17.8	15.2	12.5	16.9	10.1	18.9^**^	10.2	19.1^**^	8.0	19.7^**^
CGTAA	4.9	14.0^**^	0.0	13.4^**^	10.8	11.6	7.1	12.5	5.0	13.9^**^	7.4	13.8^*^	7.1	14.1^*^	8.0	13.2
CGTAG	6.1	3.8	11.8	3.2^**^	4.3	4.4	7.1	3.8	5.0	4.2	1.9	5.9^*^	2.2	5.8^*^	4.0	4.7
TACAG	9.9	0.4^**^	14.4	1.2^**^	4.5	2.8	7.3	2.0^**^	8.8	0.9^**^	4.3	2.4	3.7	2.7	2.6	3.3
CACGG	0.1	3.9^*^	0.1	3.3^**^	0.1	3.4^**^	0.1	3.5^*^	0.1	3.9^*^	4.7	1.8^*^	5.4	1.3^**^	6.0	1.2^**^
CGCAG	3.7	1.0^*^	7.2	0.8^**^	0.0	2.0	0.0	2.1	2.5	1.4	1.9	1.6	1.8	1.7	2.0	1.6

* *P* < 0.05,

** *P* < 0.01

Our haplotype analysis showed that recipients with the CACAG and CGTAA haplotypes had better long-term kidney function (36 months after renal transplantation) based on eGFR [17] (*P <* 0.01). On the other hand, the TACGG haplotype was associated with poorer kidney function 24 or 36 months after renal transplantation (*P <* 0.05 or *P* < 0.01). The frequencies of the CGTAG, TACAG, CACGG and CGCAG haplotypes were very low or no association with long-term eGFR was observed.

## DISCUSSION

CTLA-4 is a costimulatory receptor that controls T-cell activation. Its’ fusion protein was approved by the U.S. Food and Drug Administration in June 2011 for the prophylaxis of organ rejection in adult patients receiving a kidney transplant [18]. Kusztal M et al. found that rs231775 was associated with long-term kidney allograft function in Caucasian recipients [19]. In the present study, we assessed the influence of five *CTLA-4* SNPs on long-term kidney function after renal transplantation in Chinese people. We observed that over a 60-month period after renal transplantation, eGFR was higher in individuals bearing the rs733618C, rs3087243A and rs5742909TT genotypes and lower in those with the rs733618TT, rs3087243GG and rs231775GG genotypes. These results are consistent with earlier reports that *CTLA-4* rs231775 and rs3087243 are associated with long-term kidney allograft function or over survival [5, 19]. However, the present study is the first to report that *CTLA-4* rs733618 SNP is associated with long-term kidney function after renal transplantation. Interestingly we also found in an earlier study that rs733618 is associated with AR after renal transplantation [10]. This may reflect ethnic differences between Asians and Caucasians, in whom the frequencies of the T allele are 60% and 93-94%, respectively [20]. Regarding the *CTLA-4* rs5742909 SNP, the potential benefit was very weak (*P* = 0.049) and will require further examination of a larger sample to confirm.

Recombinant CTLA-4 containing a Thr17Ala substitution caused by the +49A/G (rs231775) SNP showed significantly less ability to inhibit T-cell proliferation and activation than its counterpart CTLA-4-17Thr [21, 22]. This means a stronger immune response is achieved with the rs231775G/GG genotype. Consequently, as we’ve observed previously [9, 10], the rs231775 SNP is associated with a higher incidence of AR and worse allograft function after renal transplantation. The CTLA-4 rs3087243 SNP could decrease sCTLA-4 levels at the cellular level and in renal transplant recipients [23, 24]. However, lower sCTLA-4 levels lead to weaker immunosuppression [23], which may explain the poorer kidney function in recipients with the rs3087243 G/GG genotype. The *CTLA-4* rs733618 SNP is located in the promoter region and reduces binding of the transcription factor nuclear factor 1 to its binding site, thereby weakening expression of cell surface CTLA-4 [25]. This in turn decreases suppression of immune attacks after renal transplantation. It is therefore not surprising that recipients carrying the T/TT genotypes at *CTLA-4 rs*733618 have a higher incidence of AR and poorer renal function than those carrying the *CTLA-4* rs733618 C/CC+CT genotypes [10].

Consistent with the genotype and allelic analyses, the haplotypes of the aforementioned *CTLA-4* SNPs and were associated with the eGFR and long-term kidney function in the transplant recipients. While the TACGG haplotype, which includes rs733618T, rs231775G and rs3087243G, was associated with poorer kidney function, the CACAG (including rs733618C, rs231775A and rs3087243G) and CGTAA (including rs733618C, rs231775A and rs3087243A) haplotypes were associated with higher eGFRs and better kidney function. It appears, therefore, that the *CTLA-4* rs733618 and rs231775 SNPs are potentially useful predictors of long-term allograft function after renal transplantation.

In conclusion, our results show that the favorable rs733618C, rs3087243A and rs5742909TT genotypes and CACAG and CGTAA haplotypes may serve as indicators of better allograft function in Chinese renal transplantation recipients. At the same time, patients with the rs733618TT, rs3087243GG and rs231775GG genotypes and the TACGG haplotype may have poorer long-term kidney function. These results provide new clues for individualized immunotherapy, especially long-term therapy for renal transplant recipients, though they will require confirmation by larger studies.

## MATERIALS AND METHODS

### Patients

The study was conducted in China and approved by the Ethics Committee of Shanghai Jiao Tong University. All participants provided written informed consent. A total of 292 cadaveric renal transplantation recipients (194 men and 98 women; median age, 46.0 years) were included. The transplantations took place at the Shanghai Organ Transplantation Center from 2006 to 2009 with follow-ups of not less than 60 months. The blood groups of all recipients were matched with their donors, as were a panel of reactive antibody and HLA-A-B-DR. Blood samples were collected from all patients for genetic analysis at the start of the study and for the evaluation of the creatinine concentration 1, 3, 6, 12, 24, 36, 48 and 60 months after renal transplantation. The eGFR was calculated using the abbreviated Modification of Diet in Renal Disease (MDRD) study equation, c-aGFR(ml/min per 1.73m^2^) = 186×[sCr (mg/dL)]-1.154 × [age(years)]-0.203 × (0.742 if female)×(1.233 if Chinese) [26].

### Immunosuppression protocol

Mycophenolate mofetil (MMF) and methylprednisolone were given prior to surgery and on the 2 days after the operation. A standard immunosuppressive protocol of triple therapy with Cyclosporine (CsA)/Tacrolimus (TAC), MMF and prednisone was administered beginning on the third day after the operation. The MMF dosage was 1.0-1.5 g/day with a weight of 60 kg as the critical value. The CsA and TAC were started at dosages of 8 mg/kg/day and 0.2 mg/kg/day, respectively, and then adjusted based on the blood drug concentration and serum creatinine.

### Sample collection and polymorphism genotyping

*CTLA-4* SNPs (*CTLA-4* rs733618, rs4553808, rs5742909, rs231775 and rs3087243) were genotyped using the method described previously with polymerase chain reaction (PCR) and direct sequencing [10].

### Statistical analysis

Statistical analysis was performed using SPSS 20.0 software (SPSS Inc., Chicago IL, USA). The eGFR in those renal recipients were compared between genotype groups using the nonparametric Kruskal-Wallis test followed by the Mann-Whitney test. An adjusted *P* value < 0.05 was considered statistically significant. Genotype associations were analyzed using a dominant model (minor-allele homozygotes plus heterozygotes vs. major-allele homozygotes) and a recessive model (minor-allele homozygotes vs. heterozygotes plus major-allele homozygotes). The allelic frequencies were counted in a single strand of measured DNA. The effects of SNPs, time and the SNPs*time interaction on eGFR were determined using repeated-measures ANCOVA. We explored the haplotype association for five SNPs using Haploview version 4.2. Haplotype was analyzed using the expectation-maximum algorithm.
